# Ocular Manifestations of COVID-19: A Systematic Review and Meta-analysis

**DOI:** 10.18502/jovr.v16i1.8256

**Published:** 2021-01-20

**Authors:** Naser Nasiri, Hamid Sharifi, Azam Bazrafshan, Atefeh Noori, Mohammad Karamouzian, Ali Sharifi

**Affiliations:** ^1^HIV/STI Surveillance Research Center, and WHO Collaborating Center for HIV Surveillance, Institute for Futures Studies in Health, Kerman University of Medical Sciences, Kerman, Iran; ^2^Neuroscience Research Center, Institute of Neuropharmacology, Kerman University of Medical Sciences, Kerman, Iran; ^3^Department of Health Research Methods, Evidence, and Impact, McMaster University, Hamilton, ON, Canada; ^4^School of Population and Public Health, Faculty of Medicine, University of British Columbia, Vancouver, BC, Canada; ^5^Department of Ophthalmology, Shafa Hospital, Afzalipour School of Medicine, Kerman University of Medical Sciences, Kerman, Iran

**Keywords:** Conjunctivitis, COVID-19, Meta-analysis, Ocular Manifestations, Systematic Review

## Abstract

Several studies have reported the characteristics of Coronavirus disease 2019 (COVID-19), yet there is a gap in our understanding of the ocular manifestations of COVID-19. In this systematic review and meta-analysis, we investigated the prevalence of ocular manifestations in COVID-19 patients. We searched Pubmed, Embase, Scopus, Web of Science, and medRxiv from December 1, 2019 to August 11, 2020. Two independent reviewers screened the articles, abstracted the data, and assessed the quality of included studies in duplicate. Thirty-eight studies were eligible after screening of 895 unique articles, with a total of 8,219 COVID-19 patients (55.3% female; *n* = 3,486 out of 6,308 patients). Using data extracted from cross-sectional studies, we performed random-effects meta-analyses to estimate the pooled prevalence of ocular symptoms along with 95% confidence interval (CI). The prevalence of ocular manifestations was estimated to be 11.03% (95% CI: 5.71–17.72). In the studies that reported the details of observed ocular symptoms, the most common ocular manifestations were dry eye or foreign body sensation (*n* = 138, 16%), redness (*n* = 114, 13.3%), tearing (*n* = 111, 12.8%), itching (*n* = 109, 12.6%), eye pain (*n* = 83, 9.6%) and discharge (*n* = 76, 8.8%). Moreover, conjunctivitis had the highest rate among reported ocular diseases in COVID-19 patients (79 out of 89, 88.8%). The results suggest that approximately one out of ten COVID-19 patients show at least one ocular symptom. Attention to ocular manifestations, especially conjunctivitis, can increase the sensitivity of COVID-19 detection among patients.

##  INTRODUCTION

Severe acute respiratory syndrome coronavirus 2 (SARS-CoV-2) was initially detected in late 2019 in Wuhan, China,^[1]^ and Coronavirus disease 2019 (COVID-19) swiftly spread across the globe, and was declared a pandemic on March 11, 2020.^[2]^ By August 14, 2020, 21,092,096 people were infected with SARS-CoV-2, 757,727 of whom passed away due to COVID-19 or its adverse health consequences.^[3]^

COVID-19 may pose challenges in clinical diagnosis because there is no pathognomonic symptom to detect the disease. Several clinical symptoms have been frequently reported among COVID-19 patients including but not limited to cough, fever, fatigue, sore throat, nasal obstruction, shortness of breath, headache, sputum production, and hemoptysis.^[4]^ Moreover, while some patients show a wider range of gastrointestinal symptoms such as diarrhea, abdominal pain, low appetite, and vomit,^[5]^ others have shown renal and ocular symptoms.^[6]^

Most clinical research about SARS-CoV-2 have focused on respiratory manifestations; however, a growing body of evidence has raised concerns about the ocular complications caused by SARS-CoV-2.^[7]^ The reported ocular manifestations of the infection vary greatly and include dry eye, foreign body sensation, itching, blurring of vision, conjunctivitis, chemosis, and photophobia.^[8]^ Some studies have even reported conjunctivitis as an early sign for COVID-19 diagnosis.^[9]^ Knowing the prevalence and type of ocular manifestations of COVID-19 can help physicians diagnose the infection better and sooner in the course of the disease. Therefore, we aimed to summarize the relevant published literature on the ocular manifestations of the COVID-19 patients.

##  METHODS

We completed our systematic review in accordance with the preferred reporting items for systematic reviews and meta-analyses (PRISMA) guideline (See Supplementary file S1 for PRISMA checklist).^[10]^

For this systematic review and meta-analysis, we searched Pubmed, Embase, Scopus, Web of Science, and medRxiv preprint server from December 1, 2019 to August 11, 2020 for studies published in English (See Supplementary file S2 for a sample search strategy). We also searched the reference lists of related systematic reviews for potentially eligible studies.

### Inclusion Criteria and Study Selection

We included empirical observational studies including cohort, case-control, cross-sectional, case-reports, or case-series that reported about ocular manifestations in COVID-19 patients. We excluded editorials, commentaries, letters to editors, and reviews. Two reviewers (NN and HSH) independently, and in duplicate, screened the titles and abstracts of identified citations, and assessed the full-text of potentially eligible studies for inclusion in the data synthesis. The reviewers resolved the disagreements on the process of study selection through feedback and discussion with the senior author (ASH).

### Data Collection

Two authors (NN and AB) independently, and in duplicate, extracted data from each eligible study, including study characteristics (e.g., first author, publication date, study type, location, and total sample size) and patients' information (e.g., age, sex, and ocular manifestations such as conjunctival hyperemia, clear secretions, conjunctivitis, follicles, petechia, and chemosis).

### Quality Assessment of the Evidence 

Two independent reviewers evaluated the quality of included studies duplicate using the Joanna Briggs Institute critical appraisal tool.^[11]^ The criteria suggested by Joanna Briggs to assess quality include eight items for case-report studies, nine items for cross-sectional studies, and ten items for case-series. Reviewers resolved the disagreements by adjudication or feedback from the senior author.

### Statistical Analysis

Data were presented using descriptive statistics (i.e., mean, median, and standard deviation [SD] for continuous variables and frequency and percentage for categorical variables). To assess the proportion of patients with a particular manifestation, we calculated the sum of the patients with a particular manifestation in different papers and divided them to the number of included patients. To account for the different study designs included in the study, we only considered cross-sectional studies in our meta-analysis. Using random-effects meta-analysis, we calculated the pooled estimated prevalence and 95% confidence interval (95% CI) of ocular manifestations, using *metaprop* command in Stata version 14.2. We also assessed the heterogeneity among the included studies using I${}^{2}$ and the Q-statistic. A value of ≥50% of I${}^{2}$ and a *P*-value of <0.1 for the Q-statistic was perceived as considerable heterogeneity. We then ran a meta-regression to assess the potential sources of heterogeneity. The following variables were included in the meta-regression: Method of COVID-19 diagnosis (polymerase chain reaction [PCR] or computed tomography scan [CT scan] vs clinical signs), the quality of studies (quality score < 4 vs quality score ≥ 4), the mean age of patients (age ≤ 45 years vs age > 45 years), the method of examination by ophthalmologist (standard ophthalmic exam vs non-standard ophthalmic exam), and the recruited sample size (sample size > 500 vs sample size ≤ 500). Based on the reported information in the papers, we also aimed to assess whether the reported ocular manifestations preceded or followed the presence of systemic symptoms. To do so, we calculated the lag between ocular manifestation and systemic disease as well as the lag between systemic disease and ocular manifestation. All statistical analyses were performed in Stata version 14.2 and all comparisons were two-tailed, with a threshold *P*-value**of 0.05.

##  RESULTS

**Figure 1:**
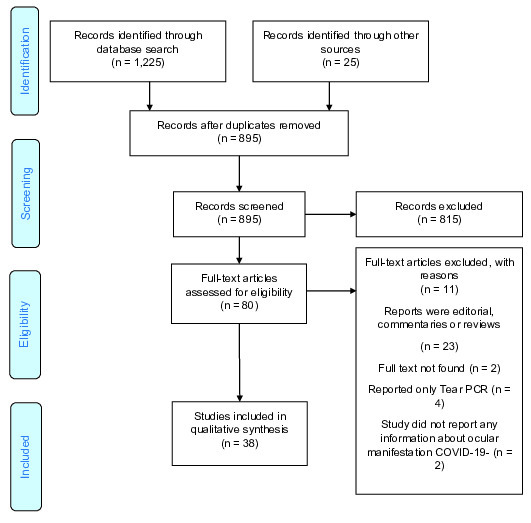
Flowchart of studies included in the systematic review of COVID-19 ocular manifestation

Out of the 895 unique publications that were assessed, 38 studies^[12,13,14,15,16,17,18,19,20,21,22,23,24,25,26,27,28,29,30,31,32,33,34,35,36,37,38,39,40,41,42,43,44,45,46,47,48,49]^ were included in this review (Figure 1). Overall, 13 studies were case reports,^[37,38,39,40,41,42,43,44,45,46,47,48,49]^ six were case-series study,^[13,15,18,25,28,36]^ and the remaining 19 studies were cross-sectional.^[12,14,16,17,19,20,21,22,23,24,26,27,29,30,31,32,33,34,35]^ Twenty-four studies reported aggregate-level^[12,13,14,15,16,17,18,19,20,21,22,23,24,25,26,27,28,29,30,31,32,33,34,35]^ and fourteen^[36][37,38,39,40,41,42,43,44,45,46,47,48,49]^reported individual-level information about ocular manifestations. Out of the 38 studies, 1 study^[16]^ was conducted among healthcare providers (see Supplementary file S3 for type of study, sex, mean age, and main ocular manifestations; Supplementary file S4 for location, publication data, patient population, and chronic disease). Moreover, out of the 38 included studies, 32 (3,719 out of 8,219 patients) were among inpatients, four among outpatients (2,353 out of 8,219 patients), and two included outpatient and inpatient individuals, simultaneously (2,147 out of 8,129 patients).

**Figure 2:**
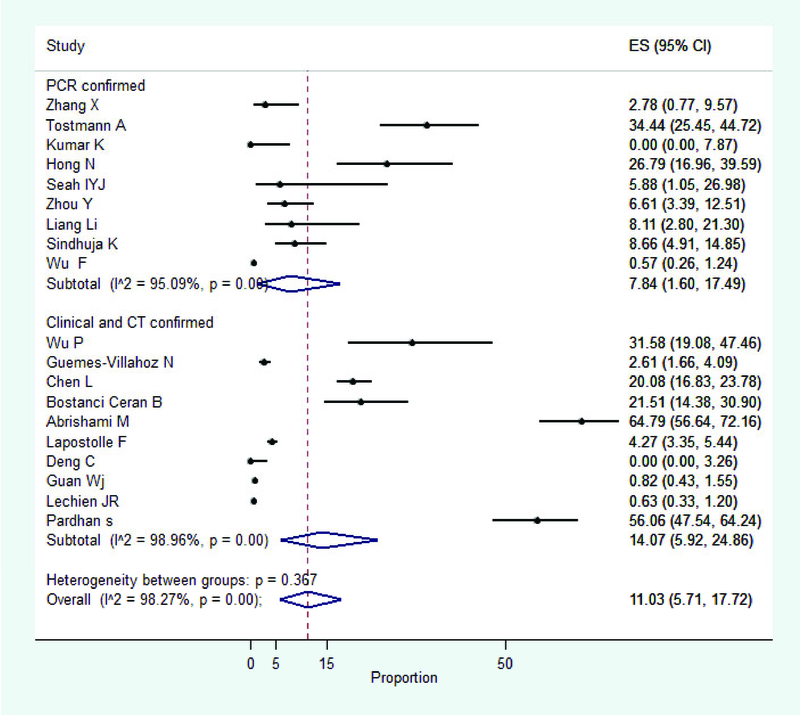
Pooled prevalence of ocular manifestation among patients with COVID-19

Demographic and clinical characteristics of COVID-19 patients included in the reviewed studies are presented in Table 1. A total of 8,219 patients with COVID-19 were enrolled in the included studies. Across all COVID-19 studies, 6,308 reported sex distribution, 1,532 reported other comorbidities with COVID-19, and 1,021 were at the individual level and reported ocular symptoms and signs. The number of enrolled patients in the included studies ranged from 1 to 1,452, most patients were female (*n* = 3,486 out of 6,308 patients, 55.3%), and the mean age of the participants ranged between 7 and 65.8 years. The diagnosis of SARS-CoV-2 was confirmed in 4,039 (49.1%) and 4,180 (50.9) patients using clinical signs and CT scans. The most detected comorbidities in patients were hypertension (593 out of 1,532), diabetes mellitus (294 out of 1,532), respiratory diseases (219 out of 1,532), and cardiovascular and cerebrovascular diseases (188 out of 1,532).

**Table 1:** Demographic and clinical characteristics of COVID-19 infection included in the reviewed studies


**Characteristics**	**** ***N*** ** (%)**
**Diagnostic approach (** ***n*** ** = 8,219) **	
Only clinical signs and CT Scan	4,180 (50.9)
PCR laboratory confirmed	4,039 (49.1)
**Sex (** ***n*** ** = 6,308)**	
Male	2,822 (44.7)
Female	3,486 (55.3)
**Comorbidity with COVID-19 (** ***n*** ** = 1, 532)**	
Hypertension	593 (38.7)
Diabetes	294 (19.2)
Respiratory system disease	219 (14.3)
Cardiovascular and cerebrovascular diseases	188 (12.3)
Cancer	60 (3.9)
Disease of immune system	59 (3.9)
Hepatitis	54 (3.5)
Liver disease	33 (2.1)
Kidney disease	32 (2.1)

**Table 2:** Symptoms and diseases of ocular in COVID-19 infection included in the reviewed studies (*n* = 1,021)


**Characteristics**	**** ***N*** ** (%)**
**Symptom and sign (** ***n*** ** = 932)**	
Dry eyes or foreign body sensation	138 (16.0)
Redness	114 (13.3)
Tearing	111 (12.8)
Itching	109 (12.6)
Eye pain	83 (9.6)
Discharge	76 (8.8)
Blurred vision or decreased vision	71 (8.2)
Photophobia	62 (7.2)
Chemosis	42 (4.9)
Irritation	21 (2.4)
Gritty feeling	14 (1.6)
Burning sensation	8 (0.9)
Lid edema	8 (0.9)
Subconjunctival hemorrhage	3 (0.3)
Pseudomembrane and hemorrhage	2 (0.2)
Pseudodendrite	1 (0.1)
Subepithelial infiltrates	1 (0.1)
Water secretion	1 (0.1)
**Disease (** ***n*** ** = 89)**	
Conjunctivitis	79 (88.8)
Keratitis	2 (2.2)
Episcleritis	2 (2.2)
Keratoconjunctivitis	2 (2.2)
Pingueculitis	1 (1.1)
Hordeolum	2 (2.2)
Posterior ischemic optic neuropathy	1 (1.1)

**Table 3:** Meta-regression analysis of the effect of the factors on the ocular manifestations of the COVID-19 patients


**Variables**	**Multivariable meta-regression**		
	**Coefficient**	*P* **-value**	**[95% conf. Interval]**
Quality of the included papers (quality ≥4 vs quality < 4)	0.02	0.59	–0.07 – 0.11
The mean age of the patients (≤ 45 years vs > 45 years	–0.11	0.29	–0.35 – 0.13
Clinical examination (standard ophthalmic exam vs non-standard ophthalmic exam	0.12	0.33	–0.17 – 0.42
Diagnostic method (PCR vs CT Scan and clinical signs)	–0.22	0.09	–0.50 – 0.05
The recruited sample size (sample size > 500 vs sample size ≤ 500)	–0.22	0.13	–0.52 – 0.09
conf., confidence			

### Quality Assessment of Included Studies

Joanna Briggs Institute's critical appraisal scores ranged from 2 to 6 for case reports (out of 8 possible points), and 0 to 5 for prevalence (cross-sectional) studies (out of 9 possible points), and 3 to 7 (out of 10 possible points) for single case-series included in the review. Quality assessment tools were different based on study design; therefore, scores could not be directly compared (See Supplementary file S5).

### The Pooled Prevalence of Ocular Manifestations

We included 19 cross-sectional studies corresponding to 7,300 individuals for meta-analysis of ocular manifestations among patients with COVID-19. The pooled prevalence of all ocular manifestations among COVID -19 patients was 11.03% (95% CI: 5.71 to 17.72) (Figure 2), The most prevalent ocular manifestations were dry eye or foreign body sensation (*n* = 138, 16.0%), redness (*n* = 114, 13.3%), tearing (*n* = 111, 12.8%), itching (*n* = 109, 12.6%), eye pain (*n* = 83, 9.6%), and discharge (*n* = 76, 8.8%). The most prevalent ocular disease was conjunctivitis (*n* = 79, 88.8%). Other rare conditions such as keratitis (*n* = 2, 2.2%), episcleritis (*n* = 2, 2.2%), keratoconjunctivitis (*n* = 2, 2.2%), hordeolum (*n* = 2, 2.2%), pingueculitis (*n* = 1, 1.1%), posterior ischemic optic neuropathy (*n* = 1, 1.1%) were also reported (Table 2). No significant source of heterogeneity from the included variables in the meta-regression was detected (Table 3).

Five studies reported the lag between ocular manifestation and systemic disease; however, nine studies reported the lag between systemic disease and ocular manifestation. Weighted mean between onset ocular manifestations and systemic disease was 0.04 days (range, 1 to 3 days). However, weighted mean between systemic disease and ocular manifestation was 1.5 days (range, 2 to 21 days).

##  DISCUSSION

This systematic review and meta-analysis included 38 studies with a total of 8,219 COVID-19 patients. Based on the existing evidence, we found the pooled prevalence of all ocular symptoms to be 11.03% (95% CI: 5.71 to 17.72) among COVID-19 patients. Dry eye or foreign body sensation was the most common reported ocular symptoms (16.0%), followed by redness (13.3%) and tearing (12.8%). The most prevalent ocular disease was conjunctivitis (88.8%).

This study showed that approximately one out of ten COVID-19 patients included in this study showed at least one ocular manifestations. Although these manifestations may not be frequent, they should not be overlooked by physicians and ophthalmologists.^[50]^ These findings are comparable with the findings of previous studies on COVID-19 or other coronaviruses. For example, Vabret *et al* in a study in a French hospital, from November 2002 to April 2003, reported that ocular manifestations were 16.7% (3 out of 18) in patients diagnosed with human coronavirus NL63.^[51]^ Moreover, Ulhaq *et al* in a systematic review study up to April 4, 2020 reported that ocular manifestations in COVID-19 patients were 5.5%.^[52]^ The reason for ocular manifestations among patients diagnosed with COVID-19 and other coronaviruses could be related to the presence of ACE2 receptor, the cell receptor for coronaviruses and SARS-CoV-2, in the eye cells.^[8]^ Transmission of SARS-CoV-2 by tear is not unlikely,^[53]^ and the eye can be a way for entering the infection droplets to the body.^[54]^ Therefore, protecting eyes is essential for people, especially for healthcare providers to protect themselves against SARS-CoV-2.

The most important ocular manifestations in COVID-19 patients were dry eye or foreign body sensation, redness, tearing, itching, eye pain, and discharge. The mechanism of dry eye or foreign body sensation is unclear in COVID-19 patients and may not be directly associated with SARS-CoV-2. Indeed, the occurrence of dry eye during the COVID-19 epidemic could be due to wearing face masks and directing the expiratory air current toward eyes, especially when masks are loose against the face and nose. The stream of air against ocular surface causes accelerated evaporation of the tear and may create dry eye symptoms. In persons with pre-existing dry eye or poor-quality tear film, the symptoms can be more common and prominent. Limitation of access to lubricating agents in fear of contamination of hands and drug containers also deteriorates dry eye manifestations.^[55,56]^ Furthermore, since the beginning of the pandemic, people spend more time looking at screens that may exacerbate dry eye sensation.^[57,58]^ While screen watching, the rate and intensity of blinks is significantly diminished, exacerbating the dry eye symptoms. Loss of follow-up visits and reduced seeking care in patients with previous dry eye condition could be other factors that may have contributed to increased dry eye symptoms during the pandemic.^[55,56]^

Conjunctivitis was the most common eye disease in patients. Conjunctivitis could be developed by certain viruses (e.g., *Haemophilus influenzae *and* Herpes simplex*), bacteria (e.g., *Staphylococcal *species*, Streptococcus pneumoniae*, and* Neisseria gonorrhoeae*), and allergies (e.g., pollen and animal dander).^[59]^ Conjunctivitis could also be developed by coronavirus and SARS-CoV-2.^[60,61]^ In a study in Iran among 142 COVID-19 patients, the most prevalent ocular finding was conjunctival hyperemia (44 persons; 31%); however, the most prevalent ocular manifestation among ICU-admitted patients was chemosis (17 out of 28 admitted to ICU; 60.7%), and 50.0% of the patients admitted to ICU (14 of the 28) showed conjunctival hyperemia.^[23]^ Scalinci *et al* in a study among five Italian COVID-19 patients reported that conjunctivitis remained through the course of the disease among COVID-19 patients.^[38]^ Hong *et al* in a study in China showed that some patients reported conjunctivitis after admission for treatment of COVID-19.^[19]^ Chen *et al *in a cross-sectional study in Wuhan China reported that some patients had conjunctivitis as their first symptom and others reported conjunctivitis after the clinical symptom of COVID-19 had begun.^[21]^ In a study in Canada, an association between conjunctivitis with corneal subepithelial infiltrations, corneal epithelial defects, development of tender preauricular lymphadenopathy, and conjunctival follicular reaction was observed among COVID-19 patients.^[44]^ Navel *et al* reported tarsal hemorrhage mucous filaments and tarsal pseudomembranous in one COVID-19 patient. They observed the eyelids were irritated by numerous sticky secretions accumulating around the eyelashes, and described mucous filaments, tarsal pseudomembranous, and superficial punctuate keratitis.^[39]^

Assessing and observing the symptoms and ocular manifestations of COVID-19 patients could improve clinicians' diagnosis of the disease. During the ongoing pandemic, ophthalmologists should consider COVID-19 as a potential diagnosis when observing ocular manifestations and conjunctivitis, especially with other manifestations of COVID-19-like respiratory signs or fever.^[60]^ Incidence of ocular symptoms may happen a few hours or days before the onset of COVID-19 systemic signs such as fever and cough.^[18,19,36]^

Ophthalmologists are at a high risk for SARS-CoV-2 given their close contact with patients. Although the transmission of SARS-CoV-2 via tear is not unlikely^[53]^ and the mechanism is uncertain,^[8,62]^ there exists a risk of transmission,^[54]^ and ophthalmologists and other healthcare providers should adhere to recommendations about wearing eye protective gears in addition to face masks and other protective devises during clinical examinations.^[63]^ This is particularly important when it comes to interactions with asymptomatic COVID-19 patients.^[1]^

We acknowledge the limitations of our study. First, ocular manifestations were measured by an ophthalmologist in some studies and through patient self-reports in others. Second, given the significant variations between the studies, we could not merge the results of different study designs. Third, most studies had a low sample size, and the quality of the included studies was low, and most were case reports and cross-sectional studies. Lastly, most COVID-19 patients are asymptomatic, but all patients enrolled in studies were symptomatic which could overestimate the infection's manifestations.

##  SUMMARY

Attention to ocular manifestations in combination with other COVID-19 manifestations could help improve COVID-19 diagnosis. The main ocular manifestations were dry eye, tearing, itching, redness, eye pain, and foreign body sensation. It is recommended that healthcare providers especially ophthalmologists who are in close contacts with patients wear eye protective goggles in addition to other recommended protective equipment.

##  Financial Support and Sponsorship

Nil.

##  Conflicts of Interest

There are no conflicts of interest.
